# Prognostic Value of the RISK-PCI Score in Patients with Non-ST-Segment Elevation Acute Myocardial Infarction

**DOI:** 10.3390/jcm14082727

**Published:** 2025-04-16

**Authors:** Ana Stanojkovic, Igor Mrdovic, Ivana Tosic, Dragan Matic, Lidija Savic, Jelena Petrovic, Andja Cirkovic, Aleksandra Milosevic, Milena Srdic, Natasa Kostic, Ivan Rankovic, Igor Petrusic

**Affiliations:** 1 Health Center Aleksinac, 18220 Aleksinac, Serbia; 2 Coronary Care Unit, Emergency Hospital, Clinical Center of Serbia, 11000 Belgrade, Serbia; igormrd@gmail.com (I.M.); dragan4m@gmail.com (D.M.); lidijasavic2007@gmail.com (L.S.); aleksandra506@gmail.com (A.M.); itana75@gmail.com (M.S.); natasabrdar99@gmail.com (N.K.); 3 School of Medicine, University of Belgrade, 11000 Belgrade, Serbia; 4 Health Center Arandjelovac, 34300 Aranđelovac, Serbia; ivanatosictasic@gmail.com; 5 Cardiology Clinic, Clinical Center of Serbia, 11000 Belgrade, Serbia; jelenapetrovic2212@gmail.com; 6 Institute for Medical Statistics and Informatics, 11000 Belgrade, Serbia; andjacirkovic01@gmail.com; 7 General Intensive Care, Emergency Hospital, Clinical Center of Serbia, 11000 Belgrade, Serbia; doctorranke@gmail.com; 8 Laboratory for Advanced Analysis of Neuroimages, Faculty of Physical Chemistry, University of Belgrade, 11000 Belgrade, Serbia; ip7med@yahoo.com

**Keywords:** acute myocardial infarction, RISK-PCI score, non-st-segment elevation, risk stratification, MACE

## Abstract

**Background:** Non-ST-segment elevation acute myocardial infarction (NSTEMI) represents a heterogeneous patient population with varying risks of adverse outcomes. The RISK-PCI score, initially developed for ST-segment elevation myocardial infarction (STEMI) patients, was evaluated for its prognostic value in NSTEMI patients undergoing percutaneous coronary intervention (PCI). **Methods:** A retrospective observational study of 242 NSTEMI patients treated with PCI at the Clinical Center of Serbia from June 2011 to June 2016 was conducted. The RISK-PCI score, incorporating clinical, echocardiographic, and angiographic variables, was calculated for each patient. The primary outcome was 30-day major adverse cardiovascular events (MACE). Secondary outcomes included individual components of MACE. Statistical analyses were performed to assess the predictive value of the RISK-PCI score. **Results:** The primary outcome of 30-day MACE occurred in 9.9% of patients. Independent predictors of 30-day MACE included age > 75 years, glucose ≥ 6.6 mmol/L, creatinine clearance < 60 mL/min, and post-procedural TIMI flow < 3. The RISK-PCI score demonstrated good discrimination for 30-day MACE (AUC = 0.725). Patients stratified into the very high-risk group (RISK-PCI score ≥ 7) had significantly higher risks of 30-day MACE (29.4%). **Conclusions:** The RISK-PCI score effectively stratifies NSTEMI patients by their risk of 30-day MACE, identifying a very high-risk subgroup that may benefit from closer monitoring and tailored interventions. External validation on larger cohorts is recommended to confirm these findings.

## 1. Introduction

Non-ST-segment elevation acute myocardial infarction (NSTEMI) represents a diverse group of patients with varying risks for death and ischemic events. Compared to patients with ST-segment elevation myocardial infarction (STEMI), those with NSTEMI tend to have more comorbidities and more extensive coronary artery disease. While some NSTEMI patients are low-risk, others are high-risk, typically older, with a higher likelihood of previous myocardial infarctions (MI) and multivessel coronary disease [1,2,3,4,5]. Due to the heterogeneity in clinical presentation and prognosis, as well as the need to determine appropriate treatment modalities, it is essential to stratify NSTEMI patients into low, medium, and high in-hospital risk groups. This stratification is achieved by using multiple risk parameters incorporated into clinical risk probability scores for predicting mortality and/or major adverse cardiovascular events (MACE) [6,7]. Among the various risk scores for NSTEMI patients, the GRACE (Global Registry of Acute Coronary Events) and TIMI (Thrombolysis in Myocardial Infarction) scores are the most commonly used [1,8]. The GRACE score has an advantage over the TIMI score in predicting mortality in NSTEMI patients, and due to its high diagnostic accuracy, it is recommended by the European Society of Cardiology for risk assessment in NSTEMI patients [9,10]. In addition to the aforementioned risk probability scores, there are also clinical probability scores for predicting cardiovascular complications or in-hospital mortality in all patients who underwent PCI (including primary PCI, urgent PCI in NSTEMI, and elective PCI for other indications). These scores were developed from the analysis of the database of the major global centers where PCI is performed [11,12].

Recent studies indicate that, despite varying risk levels, short- and long-term outcomes for NSTEMI patients have not improved significantly [4,5]. Major adverse cardiovascular events (MACE) occur frequently after percutaneous coronary intervention (PCI), with higher-risk patients experiencing worse outcomes [13,14].

A newer prognostic model, the RISK-PCI score, is used to assess post-PCI risk and identify high-risk patients who may benefit from close monitoring, early aggressive treatment, or adjustments in antiplatelet therapy. The RISK-PCI score categorizes patients into four risk groups (low, intermediate, high, and very high) and predicts short-term risks (30 days) for MACE and death in STEMI patients undergoing primary PCI (PPCI) [15]. It has also been shown to predict both early and late stent thrombosis following pPCI [16]. What distinguishes the RISK-PCI score from other risk models is its incorporation of clinical, echocardiographic, and angiographic factors, specifically designed for use in both STEMI and NSTEMI patients [8,17].

The primary hypothesis of this study is that the RISK-PCI score can help prevent death and new coronary events in NSTEMI patients, particularly with an emphasis on achieving complete revascularization before hospital discharge. A secondary hypothesis is that applying the RISK-PCI score could improve outcomes in high-risk NSTEMI patients.

This study aims to assess the prognostic value and validity of the RISK-PCI score in predicting 30-day MACE in NSTEMI patients treated with PCI, as well as identifying factors associated with an increased risk.

## 2. Materials and Methods

### 2.1. Ethical Stataement

A retrospective, observational, single-center study was conducted, analyzing 242 patients diagnosed with acute non-ST-segment elevation myocardial infarction (NSTEMI). These patients were admitted to the Emergency Center Coronary Unit at the Clinical Center of Serbia between June 2011 and June 2016. The study was approved by local ethics committees, and informed consent was obtained from all participants.

### 2.2. Patient Population and Procedures

Adult patients (aged ≥ 18 years) diagnosed with NSTEMI who underwent percutaneous coronary intervention (PCI) through either angioplasty or stent implantation, and who had complete data for all the variables of the score, were included in this study.

Exclusion criteria included patients with unavailable documentation or incomplete data for the variables of the score, those who required surgical revascularization, and those who were not percutaneously revascularized due to reasons such as allergy to DAPT, active bleeding, or iodine contrast allergy. Additionally, patients who presented with heart failure (Killip IV) at admission were also excluded.

All patients received dual antiplatelet therapy (DAPT), along with gastric mucosal protective drugs (proton pump inhibitor-PPI or H2 receptor blockers).

PCI procedures included stent implantation, balloon angioplasty, and, when needed, tirofiban administration and thrombus aspiration. Flow grades were assessed using the Thrombolysis in Myocardial Infarction (TIMI) criteria. Renal function was assessed using the Cockcroft-Gault formula, and LVEF was measured using the Simpson method in 2- and 4-chamber apical projections.

### 2.3. Data Collection and Follow-Up Protocols

Data collected from medical histories and the electronic database included the following:

– Demographic data: gender, age.
– Anamnestic data: previous myocardial infarction (MI), prior percutaneous or surgical myocardial revascularization, comorbidities (e.g., stroke, peripheral vascular disease, ulcer disease), and risk factors for coronary disease (e.g., hypertension, diabetes, hyperlipoproteinemia, smoking, obesity, body mass index—BMI), and chest pain duration (in hours).
– Physical examination data: systolic blood pressure, heart rate, Killip classification of heart failure at admission.
– Laboratory parameters: hemoglobin, leukocytes, platelets, blood glucose, creatinine clearance, fibrinogen at admission, and maximum values of troponin I and creatine kinase.
– Electrocardiographic data: arrhythmias (e.g., atrial fibrillation, complete AV block), acute left bundle branch block, infarction localization (anterior or inferior NSTEMI).
– Echocardiographic data: left ventricular ejection fraction (LVEF).
– Coronary angiography data: findings of three-vessel disease, left main stenosis, infarct-related artery (IRA), venous graft stenosis, IRA reference diameter, bifurcation lesions, and preprocedural occlusion (TIMI 0).
– Drug therapy data: dual antiplatelet therapy, beta-blockers, heparin, ACE inhibitors, statins, cardiotonics, diuretics, inotropes, antiarrhythmics, gastric mucoprotectants (PPI, H2 blockers), and GP IIb/IIIa receptor inhibitors.
– Intervention data: type of PCI (angioplasty or stent implantation), number of stents implanted, temporary pacemaker placement, post-procedural TIMI, stent length, drug-eluting stent (DES) use, and presence of coronary artery dissections.
– Complication data: outcomes within 30 days (e.g., reinfarction, stroke, repeated revascularization, or death) were obtained from medical records, telephone interviews, and outpatient visits.

### 2.4. Outcomes and Definitions

The primary outcome was major adverse cardiovascular events (MACE), defined as death, reinfarction, stroke, or repeated PCI within 30 days after NSTEMI. Secondary outcomes included individual components of MACE within this 30-day period. Reinfarction was defined as recurrent ischemic chest pain lasting more than 20 min, ST-segment elevation or depression, T wave inversion, or new Q waves in at least two contiguous leads, and an increase in cardiac troponin above the upper reference limit [18]. Stroke was defined as a focal or global neurological deficit lasting longer than 24 h, classified as either ischemic or hemorrhagic based on computed tomography. Diagnosis and treatment were managed by a neurologist from the Emergency Center. Repeated revascularization (re-PCI) was defined as PCI performed again for restoring blood flow to the coronary arteries after the initial PCI during the follow-up period [19].

### 2.5. The RISK-PCI Score

The RISK-PCI score was calculated for each participant based on 12 variables: patient age, previous myocardial infarction (MI), anterior MI, acute bundle branch block (BBB), high-grade atrioventricular (AV) block, laboratory parameters (leukocyte count, glycemia, and creatinine clearance (CCr)), left ventricle ejection fraction (LVEF), and angiographic findings (infarct-related artery (IRA) diameter and pre/post-procedural TIMI flow rate). The total score ranges from 0 to 20 (Table 1) [15].

### 2.6. Statistical Analysis

Categorical variables were expressed as counts and percentages, while numerical variables were presented as arithmetic mean with standard deviation or median with range (form minimum to maximum), depending on the data distribution. The normality was evaluated using Shapiro-Wilk test. The difference between defined groups (MACE vs. non-MACE) was evaluated using Student’s *t*-test or Mann–Whitney U test for numerical, whereas chi-square test or Fisher’s exact test was used for categorical variables. Risk factors associated with 30-day MACE were evaluated by univariate and multivariate logistic regression analysis with OR (odds ratio), 95% CI OR (95% confidence interval of the odds ratio), and *p* value reported with collinearity evaluation by correlation matrix and VIF method. The discriminating performance of the RISK-PCI score was expressed as an area under the receiving operator characteristics (ROC) curve, sensitivity (Sn), and specificity (Sp). Cut-off value was defined according to Youden’s rule. Sensitivity and specificity were chosen in the way that corresponds to the highest Youden’s index (Youden’s index = Sn + Sp − 100). All statistical methods were considered significant if the p value was equal or lower than chosen error of the first type of 0.05. The analysis was performed in statistical software IBM SPSS ver. 26 while R software (https://www.r-project.org/, (accessed on 8 April 2025)) environment was used for graphical presentations.

## 3. Results

### 3.1. Patients Characteristics

A total of 242 patients were included in the study, with a mean age of 64.39 ± 12.20 years and a male-to-female ratio of 2:1. Baseline characteristics of all patients, both with and without MACE are shown in Table 2. Patients with MACE were significantly older, predominantly male, non-smokers, and had a history of atrial fibrillation, but were less likely to have hyperlipidemia. Additionally, they had significantly lower systolic blood pressure, LVEF, and CCr, while their glycemia and peak troponin levels were significantly higher compared to those without MACE. A greater proportion of MACE patients had Killip class 2/3, 3-vessel disease, and a final TIMI flow grade < 3, compared to the non-MACE group. Furthermore, patients with MACE were more frequently treated with diuretics, antiarrhythmics, and inotropes, while non-MACE patients were more likely to receive beta-blockers (Table 3 and Table 4). The RISK-PCI score was significantly higher in patients with MACE compared to those without MACE (Table 5).

### 3.2. Outcomes

The primary outcome of major adverse cardiovascular events (MACE) occurred in 24 (9.9%) patients during the 30-day follow-up. Secondary outcomes, including death, reinfarction, cerebrovascular insult, and re-PCI, occurred in nine (37.5%), five (20.8%), three (12.5%), and five (20.8%) patients, respectively (Table 5).

### 3.3. Validation of a Score for Predicting 30-Day MACE in NSTEMI Patients

**Figure 1:**
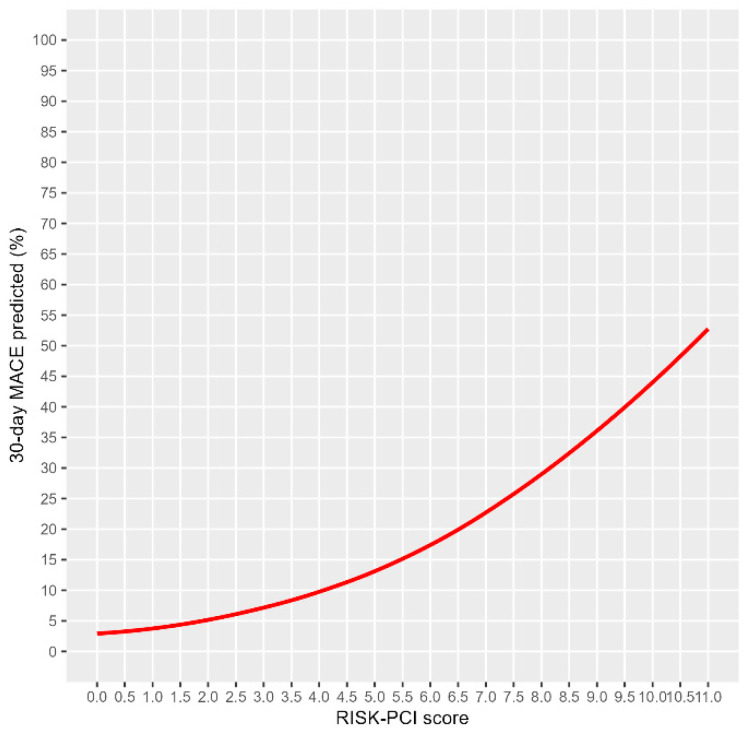
Predicted probability of 30-day MACE in NSTEMI patients corresponding to total risk points.

Factors potentially associated with 30-day MACE in NSTEMI patients included age over 75 years, glucose level ≥ 6.6 mmol/L, creatinine clearance below 60 mL/min and post-procedural IRA flow rate (TIMI < 3) (Table 6). Additionally, the total RISK-PCI score was found to correlate with 30-day MACE. Multivariate logistic regression, utilizing the Backward Wald method, identified two independent predictors of 30-day MACE in NSTEMI patients in the third analysis step: a glucose level over 6.6 mmol/L (OR = 4.558, 95% CI: 1.57–13.21, *p* = 0.005) and creatinine clearance below 60 mL/min (OR = 3.978, 95% CI: 1.52–10.40, *p* = 0.005). These associations were moderate, and the predicted probability of 30-day MACE based on the total risk score is illustrated in Figure 1. The same parameters remained independently associated with MACE even after adjusting the logistic regression model for baseline characteristics that differed (such as gender, smoking, and hyperlipidemia).

**Figure 2:**
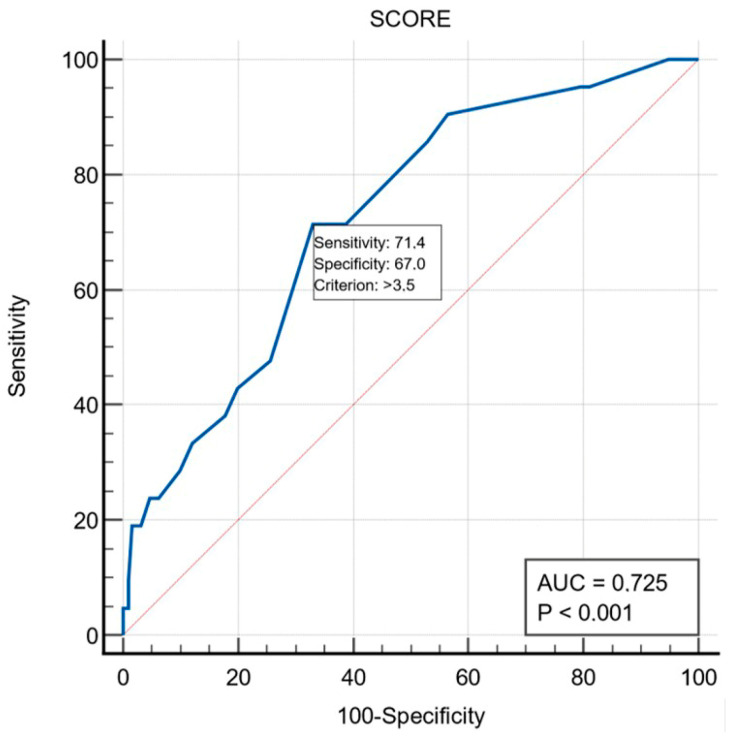
Receiving-operating characteristic curves showing the discriminating ability of the RISK-PCI score with the cut-off value of ≥3.5 for the prediction of 30-day MACE in NSTEMI patients. AUC, area under the curve.

The ability of the RISK-PCI score with the cut-off value of ≥3.5 for the prediction of 30-day MACE in patients with NSTEMI is presented in Figure 2. The area under the curve (AUC) was 72.5% (*p* < 0.001), while sensitivity and specificity were 71.4% and 67.0% for 30-day MACE.

### 3.4. Predictive Performance of the RISK-PCI Score Compare to GRACE Score

**Figure 3:**
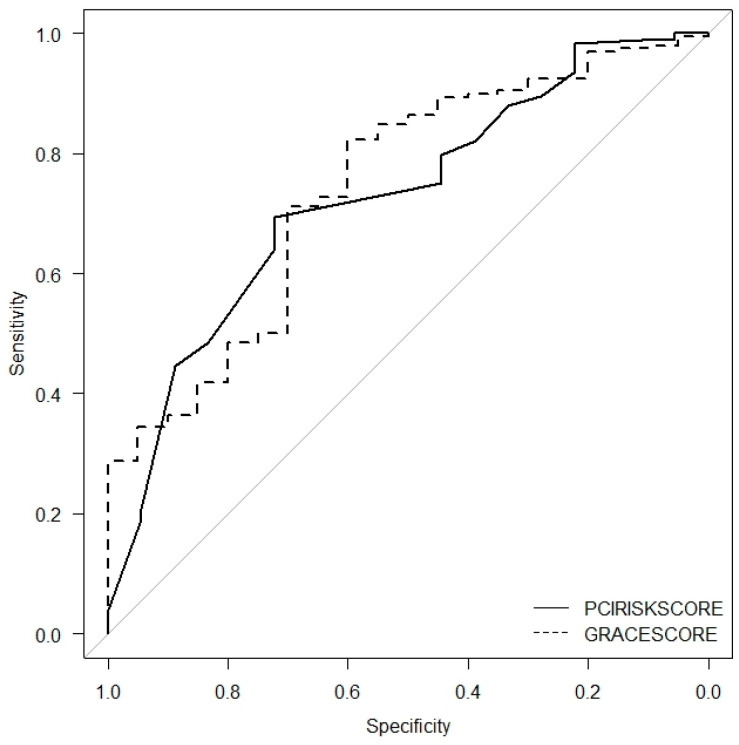
Predictive performance of the RISK-PCI score compared to GRACE score.

GRACE score and RISK-PCI score were equally powerful for predicting MACE (AUC for GRACE score = 72.6% vs. AUC for RISK-PCI score = 76.3%, *p* = 0.544) (Figure 3).

### 3.5. Risk Stratification

RISK-PCI score was stratified into four classes: low (0–2.5), intermediate (3–4.5), high (5–6.5), and very high (≥7) with the incidence of the 30-day MACE of 3.2%, 12.5%, 13.3%, and 29.4%, respectively (Table 7). Risk class with the best prediction power of 30-day MACE was very high risk with RISK-PCI score of ≥7.

## 4. Discussion

### 4.1. Key Findings

Our study found that 30-day major adverse cardiovascular events (MACE) occurred in 24 (9.9%) of NSTEMI patients who underwent percutaneous coronary intervention (PCI). Independent predictors of MACE included age over 75 years, glucose levels ≥ 6.6 mmol/L, and creatinine clearance (CCr) < 60 mL/min at admission. MACE was also associated with a final Thrombolysis in Myocardial Infarction (TIMI) flow below 3. Receiver operating characteristic (ROC) analysis demonstrated good discrimination ability of the RISK-PCI score for predicting 30-day MACE. Compared to the GRACE score, RISK-PCI score was equally as effective at predicting MACE. The highest predictive power for MACE was observed in patients categorized as very high-risk with a RISK-PCI score ≥ 7.

### 4.2. Age as a Risk Factor

Age over 75 years emerged as an independent predictor of 30-day MACE. Elderly patients with NSTEMI face higher risks of adverse cardiovascular events and complications related to treatment compared to younger individuals. Van Den Broek et al. conducted a multicenter prospective study revealing that MACE and major bleeding were more frequent in NSTEMI patients aged ≥75 years. This increased risk in elderly patients is likely multifactorial, involving age-related physiological changes and the accumulation of cardiovascular risk factors and comorbidities [20,21,22].

### 4.3. Hyperglycemia and MACE

Our study also found that glucose levels ≥ 6.6 mmol/L independently predicted 30-day MACE. A multivariable analysis by Hao et al., similarly identified admission hyperglycemia as an independent predictor of both 30-day and 3-year MACE in NSTEMI patients undergoing PCI, regardless of diabetes mellitus (DM) status. A meta-analysis of 19 studies confirmed the strong association between abnormal glucose tolerance and poor outcomes, including all-cause mortality, recurrent MACE, cardiovascular death, and hospitalization for heart failure. Hyperglycemia likely impacts prognosis through mechanisms such as endothelial dysfunction, platelet aggregation, inflammation, and oxidative stress [23,24,25].

### 4.4. Renal Function and MACE

In our study, CCr < 60 mL/min at admission was an independent predictor of MACE at 30-day follow-up. Renal failure is common in NSTEMI patients, and several studies have shown a consistent association between renal failure and the increased risk of ischemic events [26,27].

### 4.5. Final TIMI Score and MACE

A final TIMI below 3 was associated with 30-day MACE. In a study of 2767 NSTEMI patients from the Polish Registry of acute coronary syndrome (ACS), Karvovski et al. demonstrated that mortality rates were significantly higher in patients with TIMI 0–1 and 2 compared to those with TIMI 3. Achieving a final TIMI 3 in the infarct-related artery (IRA) was found to improve outcomes in NSTEMI patients treated with PCI [28].

### 4.6. RISK-PCI Score and Prognostic Value

The RISK-PCI score, which incorporates the aforementioned risk factors, enhances predictive accuracy for 30-day MACE in NSTEMI patients. This is the first study to evaluate the RISK-PCI score in patients with NSTEMI. The score demonstrated good discriminative ability for predicting 30-day MACE in NSTEMI patients (AUC = 0.725). Compared to the GRACE score, the RISK-PCI score is at least as effective, if not better, due to its inclusion of a broader range of variables that cover different aspects of risk. Our study demonstrated that both the GRACE score and the RISK-PCI score were highly effective in predicting MACE, with the RISK-PCI score showing a slightly higher predictive power (AUC for GRACE score = 72.6%, AUC for RISK-PCI score = 76.3%, *p* = 0.544). This finding further confirms that the RISK-PCI score can be a valuable tool in predicting prognosis in NSTEMI patients. In a study comparing the prognostic performance of three major scoring systems, GRACE, TIMI, and RISK-PCI, the RISK-PCI score demonstrated strong discrimination and was non-inferior to both the GRACE and TIMI scores in predicting 30-day MACE and mortality [8]. Notably, it was the only score capable of predicting recurrent ischemia that required immediate target vessel revascularization [8]. These findings are not surprising, as the RISK-PCI score was specifically designed to predict MACE, whereas the GRACE score was not originally intended for MACE prediction [29]. In our study, the RISK-PCI score demonstrated strong discriminative ability for patient stratification into low- and high-risk groups. Patients in the very high-risk group had a 12.5-fold increased risk of MACE compared to those in the low-risk group. This high-risk subgroup may benefit from extended monitoring and inpatient care. When compared to the GRACE score, both systems exhibited good prognostic value for predicting adverse cardiovascular events within the 30-day follow-up period. Specifically, one-third of patients with a RISK-PCI score ≥ 7 (very high-risk group) died during the 30-day follow-up, suggesting that such patients require close monitoring during the acute phase of myocardial infarction (MI) and careful evaluation of dual antiplatelet therapy [15]. Furthermore, high-risk patients may also be candidates for complete revascularization before discharge [15]. Additionally, neither the GRACE nor TIMI score is initially graded for the occurrence of MACE and nor do they consider angiographic and echocardiographic data, as the RISK-PCI score does. Although prognosis after acute coronary syndrome (ACS) primarily depends on the baseline risk profile, both echocardiographic and angiographic data are powerful predictors of prognosis [30].

### 4.7. Clinical Relevance of RISK-PCI Score

Our study confirms that the RISK-PCI score has clinical utility in predicting the 30-day MACE risk in NSTEMI patients. The ROC analysis indicated good predictive accuracy for this score. By incorporating both clinical and angiographic variables, the RISK-PCI score offers a more comprehensive and accurate risk stratification tool compared to scores based solely on clinical or angiographic data. This method can aid in identifying high-risk NSTEMI patients and may potentially improve outcomes by informing treatment decisions.

### 4.8. Clinical Implications

The clinical importance of assessing the risk for MACE in the 30-day follow-up period lies in identifying high-risk patients who require closer monitoring, optimization of dual antiplatelet therapy, and consideration for complete myocardial revascularization (either PCI or surgical) before hospital discharge.

### 4.9. Study Limitations

Our study has several limitations. First, the data were derived from a retrospective analysis using a single-center database. Second, patients presenting with cardiogenic shock were excluded, as these patients typically require specialized risk stratification and a different treatment approach [15]. Third, potential confounders (such as anticoagulation therapy on admission, a history of peripheral artery disease, etc.) could have influenced the results in the multivariate logistic regression analysis, but they were not considered because there were very few cases, making their inclusion irrelevant. Fourth, due to the small sample size (number of patients), a modification of the score was made by dividing it into creatinine clearance > 60 vs. <60 (eliminating the 60–90 and >90 groups), in order to reduce the impact of the small sample size on the analysis. Fifth, since the sample size is very limited, there are very large confidence intervals (CIs) for some variables. Sixth, our analysis was limited to patients with NSTEMI who underwent PCI, so the findings may not be applicable to those who were not revascularized by percutaneous intervention but required surgical revascularization. Finally, this study was designed to apply the RISK-PCI score in NSTEMI patients treated with PCI, rather than to compare the characteristics of the RISK-PCI score with those of other risk scores for patients treated with PCI, or to perform the external validation of the model.

## 5. Conclusions

This study highlights a promising new application of the RISK-PCI score, demonstrating its potential to provide valuable prognostic insights for clinical outcomes in NSTEMI patients who undergo PCI. The score effectively distinguishes a very high-risk subgroup for 30-day MACE, offering important risk stratification. However, to further assess the validity and generalizability of this model, external validation on a larger cohort of NSTEMI patients is needed.

## Figures and Tables

**Table 1:** RISK-PCI score.

Risk Factor	Points	Risk Factor	Points
		Creatinine Clearance (CCr) *	
Age > 75 years	1	≥90 mL/min	0
Previous heart attack	1.5	CCr 60–89 mL/min	1
Anterior infarction	1	CCr < 60 mL/min	2
Complete AV block *	2	LVEF < 40%	1.5
Acute BBB *	3.5	Ref. diameter ≤ 2.5 mm	1
Leukocytes * > 12.0 × 10^−9^/L	1	Previous TIMI = 0	1
Glycemia * ≥ 6.6 mmol/L	1	Final TIMI < 3	3.5

* At admission. TOTAL SCORE 0–20. AV, atrioventricular; BBB, bundle branch block; LVEF, left ventricular ejection fraction; TIMI, thrombolysis in myocardial infarction.

**Table 2:** Characteristics of all NSTEMI patients, with and without MACE.

Characteristi-cs	Total n = 242	MACE n = 24	Non-MACE n = 218	*p* *
**Baseline**				
Age (years), mean ± sd	64.39 ± 12.20	69.75 ± 14.33	63.80 ± 11.83	**0.023 £**
Age > 75 years, n (%)	56 (23.1)	11 (45.8)	45 (20.6)	**0.005 ¥**
Gender (female), n (%)	72 (29.8)	2 (8.3)	70 (32.1)	**0.016 ¥**
BMI, kg/m^3^	24.81 ± 3.21	24.65 ± 3.07	24.82 ± 3.24	0.812 £
Smoking, n (%)	129 (53.3)	8 (33.3)	121 (55.5)	**0.039 ¥**
**Medical history, n (%)**				
HTA	210 (86.8)	20 (83.3)	190 (87.2)	0.600 ¥
DM	67 (27.7)	8 (33.3)	59 (27.1)	0.515 ¥
Hyperlipidemia	173 (71.5)	13 (54.2)	160 (73.4)	**0.048 ¥**
Previous AMI	51 (21.2)	4 (16.7)	47 (21.7)	0.570 ¥
Previous CABG	26 (10.7)	3 (12.5)	23 (10.6)	0.770 ¥
Previous PCI	49 (20.2)	4 (16.7)	45 (20.6)	0.646 ¥
Previous complete AV block	3 (1.2)	1 (4.2)	2 (0.9)	0.270 €
Previous atrial fibrillation	23 (9.5)	5 (20.8)	18 (8.3)	**0.046 ¥**
Previous CVI	18 (7.4)	4 (16.7)	14 (6.4)	0.069 ¥
Previous peptic ulcer	29 (12.0)	1 (4.2)	28 (12.8)	0.214 ¥
Peripheral vascular disease	11 (4.5)	1 (4.2)	10 (4.6)	0.925 ¥
**Admission characteristics**				
SBP (mmHg), mean ± sd	140.48 ± 23.09	127.50 ± 23.82	141.90 ± 22.61	**0.004 £**
HR (beats/min), mean ± sd	78.88 ± 14.91	82.39 ± 20.34	78.50 ± 14.22	0.235 £
Peak CK, med (min–max)	274.0 (0.0–3163.0)	364.0 (16.0–3163.0)	260.0 (0.0–2897.0)	0.350 $
Peak troponin, med (min–max)	2.8 (0.1–126.1)	6.5 (0.9–126.1)	2.4 (0.1–72.8)	**0.013 $**
Hgb (g/L), mean ± sd	136.10 ± 17.44	131.83 ± 22.59	136.58 ± 16.78	0.207 £
Leukocyte (×10^9^/L), med (min–max)	9.0 (3.0–26.3)	8.9 (3.6–26.3)	9.0 (3.0–19.4)	0.824 $
Platelet count (×10^9^/L), med (min–max)	220 (20–603)	213.5 (115–330)	220.5 (20–603)	0.415 $
Fibrinogen (mg/mL), mean ± sd	4.36 ± 1.06	4.50 ± 1.14	4.34 ± 1.05	0.499 £
Glycemia (mmol/L), med (min–max)	6.2 (3.9–23.7)	7.2 (4.2–23.2)	6.1 (3.9–23.7)	**0.012 $**
Glycemia > 6.6 mmol/L, n (%)	105 (43.6)	18 (75.0)	87 (40.1)	**0.001 ¥**
Creatinine clearance (mL/min), med (min–max)	79.5 (4.0–169.0)	48.0 (6.0–115.0)	82.0 (4.0–169.0)	**<0.001 $**
Creatinine clearance (mL/min), n (%)				
<60	65 (30.1)	13 (61.9)	52 (26.7)	**0.001 ¥**
>60	151 (69.6)	8 (38.1)	143 (73.3)	
Left ventricle EF (%), mean ± sd	52.12 ± 12.24	44.83 ± 15.30	52.93 ± 11.62	**0.002 £**
Left ventricle EF < 40%, n (%)	27 (11.2)	5 (20.8)	22 (10.0)	0.113 ¥
Killip class 2/3, n (%)	30 (12.4)	9 (37.5)	21 (9.6)	**<0.001 ¥**
AIM localization, n (%)				
anterior	125 (51.7)	13 (54.2)	112 (51.4)	0.795 ¥
inferior	89 (36.8)	6 (25.0)	83 (38.1)	0.207 ¥
Pain duration before FMC, med (min–max)	8.0 (5.0–120.0)	8.0 (0.8–48.0)	8.0 (0.5–120.0)	0.598 $

* for the level of significance of 0.05 according to Student’s *t*-test £, Mann–Whitney U test $, Chi-square test ¥, Fisher’s exact test €. AMI, acute myocardial infarction; AV, atrioventricular; BMI, body mass index; CABG, coronary artery bypass grafting; CK, creatine kinase; CVI, cerebrovascular insult; DM, diabetes mellitus; EF, ejection fraction; FMC, first medical contact; HR, heart rate; HTA, arterial hypertension; MACE, major adverse cardiovascular events; NSTEMI, non-st-segment acute myocardial infarction; PCI, percutaneous coronary intervention; PPI, proton pump inhibitor; SBP, systolic blood pressure.

**Table 3:** Angiographic findings of all NSTEMI patients with and without MACE.

Angiographic Findings	Total n = 242	MACE n = 24	Non-MACE n = 218	*p* *
Pre-procedural TIMI flow grade 0, n (%)	23 (9.5)	2 (8.3)	21 (9.6)	0.837 ¥
3-vessel disease, n (%)	76 (31.4)	15 (62.5)	61 (28.0)	**<0.001 ¥**
Left main disease, n (%)	22 (9.1)	3 (12.5)	19 (8.7)	0.540 ¥
Bifurcation lesion, n (%)	19 (7.9)	1 (4.2)	18 (8.3)	0.480 ¥
Reference diameter (mm), mean ± sd	3.09 ± 0.51	3.15 ± 0.35	3.00 ± 0.52	0.585 £
Reference diameter < 2.5 mm, n (%)	41 (17.2)	1 (4.3)	40 (18.5)	0.087 ¥
Saphenous vein graft, n (%)	21 (35.0)	2 (25.0)	19 (36.5)	0.524 ¥
**Procedural** **characteristics**				
PTCA, n (%)	64 (26.4)	7 (29.2)	57 (26.1)	0.750 ¥
Tirofiban, n (%)	21 (10.5)	3 (17.6)	18 (9.8)	0.315 ¥
Stent, n (%)	182 (75.2)	19 (79.2)	163 (74.8)	0.636 ¥
Number of stents, med (min–max)	1 (0–5)	1 (0–3)	1 (0–5)	0.842 $
IRA max stent length (mm), med (min–max)	22 (12–83)	22 (15–64)	22 (12–83)	0.679 $
IRA DES, n (%)	75 (31.0)	10 (41.7)	65 (29.8)	0.233 ¥
IRA Dissection, n (%)	11 (4.5)	1 (4.2)	10 (4.6)	0.925 ¥
Final TIMI flow grade < 3, n (%)	23 (9.6)	5 (21.7)	18 (8.3)	**0.038 ¥**

* for the level of significance of 0.05 according to Student’s *t*-test £, Mann–Whitney U test $, Chi-square test ¥. IRA, infarct-related artery; MACE, major adverse cardiovascular events; NSTEMI, non-st-segment acute myocardial infarction; PTCA, percutaneous transluminal coronary angioplasty; TIMI, thrombolysis in myocardial infarction.

**Table 4:** Therapy of all NSTEMI patients with and without MACE.

Therapy During Hospitalization (in Addition to DAPT and PPI/H2), n (%)	Total n = 242	MACE n = 24	Non-MACE n = 218	*p* *
Beta blocker	204 (84.3)	16 (66.7)	188 (86.2)	**0.012 ¥**
Heparin	236 (97.5)	23 (95.8)	213 (97.7)	0.469 €
ACE inhibitor	203 (83.9)	18 (75.0)	185 (84.9)	0.212 ¥
Statin	241 (99.6)	24 (100.0)	217 (99.5)	1.000 €
Diuretic	65 (26.9)	14 (58.3)	51 (23.4)	**<0.001 ¥**
Antiaritmics	23 (9.5)	6 (25.0)	17 (7.8)	**0.006 ¥**
Inotropics	15 (6.2)	6 (25.0)	9 (4.1)	**<0.001** ¥
Cardiac glycoside	7 (2.9)	1 (4.2)	6 (2.8)	0.523 €

* for the level of significance of 0.05 according to Chi-square test ¥, Fisher’s exact test €. ACE, angiotensin-converting enzyme; DAPT, dual antiplatelet therapy; H2 blocker, histamine H2-receptor antagonist; MACE, major adverse cardiovascular events; NSTEMI, non-st-segment acute myocardial infarction; PPI, proton pump inhibitor.

**Table 5:** Outcomes of all NSTEMI patients with- and without MACE.

Outcomes	Total n = 242	MACE n = 24	Non-MACE n = 218	*p* *
Death, n (%)	9 (3.7)	9 (37.5)	0 (0.0)	**<0.001 ¥**
Re-AMI, n (%)	5 (2.1)	5 (20.8)	0 (0.0)	**<0.001 ¥**
CVI	3 (1.2)	3 (12.5)	0 (0.0)	**<0.001 ¥**
Re-PCI	5 (2.1)	5 (20.8)	0 (0.0)	**<0.001 ¥**
RISK-PCI score, med (min–max)	3 (0.0–11.5)	4 (1–11.5)	3 (0–11.0)	**0.001 $**

* for the level of significance of 0.05 according to Chi-square test ¥ and Mann–Whitney U test $. CVI, cerebrovascular insult; MACE, major adverse cardiovascular events; NSTEMI, non-st-segment acute myocardial infarction; Re-PCI, repeated revascularization with PCI; Re-AMI, reinfarction.

**Table 6:** Factors associated with 30-day MACE in NSTEMI patients (univariate analysis).

Parameter	MACE		
	OR	95%CI OR	*p* *
Age > 75 years	3.25	1.37–7.74	**0.008**
Prior infarction	0.72	0.24–2.22	0.571
Anterior infarction	1.12	0.48–2.61	0.795
Complete AV block	4.70	0.41–53.80	0.214
Acute BBB	-	-	-
Leukocyte >12 × 10^9^/L	1.01	0.36–2.86	0.982
Glucose ≥ 6.6 mmol/L	4.48	1.71–11.74	**0.002**
Creatinine Clearance < 60 mL/min	4.47	1.75–11.40	**0.002**
LVEF < 40%	2.34	0.80–6.90	0.122
IRA reference diameter ≤ 25 mm	0.20	0.03–1.53	0.121
IRA pre-procedural TIMI flow grade 0	0.85	0.19–3.88	0.837
IRA post-procedural TIMI flow grade < 3	3.06	1.02–9.20	**0.047**
RISK-PCI score	1.39	1.16–1.68	**<0.001**

* for the level of significance of 0.05. CI, confidence interval; OR, odds ratio; others, see Table 2 and Table 3.

**Table 7:** Risk stratification and 30-day MACE prediction in NSTEMI patients.

Risk Class	n (%)	Score Category	MACE (%) Observed vs. Expected	OR (95% CI RR) for 30-Day MACE
Low	93 (43.9)	0–2.5	3.2 vs. 1.1–9.1	Ref.
Intermediate	72 (34.0)	3–4.5	12.5 vs. 6.7–22.1	4.28 (1.12–16.46)
High	30 (14.2)	5–6.5	13.3 vs. 5.3–29.7	4.62 (0.97–21.95)
Very high	17 (8.0)	≥7	29.4 vs. 13.3–53.1	12.50 (2.64–59.07)

## Data Availability

The original contributions presented in this study are included in the article. Further inquiries can be directed to the corresponding author.
